# Does proper flossing performance translate into effective plaque removal?

**DOI:** 10.1007/s00784-025-06505-z

**Published:** 2025-09-04

**Authors:** Katja Jung, Björn Böttge, Mathis Kullmann, Carolina Ganss

**Affiliations:** 1 Department of Restorative Dentistry and Endodontology, Dental Clinic, Justus-Liebig-University of Giessen, Giessen, Germany; 2 Present Address: Department of Operative Dentistry, Endodontics, and Paediatric Dentistry, Section of Cariology, Medical Centre of Dentistry, Philipps-University Marburg, Marburg, Germany; 3 Present Address: Department of Prosthetic Dentistry, Medical Center - University of Freiburg, Center for Dental Medicine, Faculty of Medicine, University of Freiburg, Freiburg, Germany

**Keywords:** Dental plaque, Dental floss, Video observation, Intraoral scanning, Interdental hygiene

## Abstract

**Objectives:**

Interdental hygiene plays a crucial role in maintaining oral health, yet the effectiveness of dental floss remains a subject of debate. Thus, this study aimed to assess whether improved flossing technique relates to improved cleaning efficacy.

**Materials and methods:**

A total of 37 adults (23.1 ± 3.2 years) participated. After habitual toothbrushing, plaque was disclosed and an intraoral scan was performed; afterwards, habitual flossing was videotaped, followed by a second intraoral scan of disclosed plaque. Participants then watched an instruction video. After one week of practice, flossing was again videotaped and intraoral scans were taken before and after flossing. On defined regions on images obtained from the four scans, plaque coverage was assessed with a three-level score (0: none, 1: <50%, 3: ≥50%). Proximal Surface Plaque Index (PSPI) was calculated as mean from all scores. A flossing performance score (FPS) was generated from the percentage of interdental spaces that were correctly flossed (interdental space reached, floss correctly applied and vertical movements; 0: totally imperfect, 3: perfect flossing). Ramfjord teeth (16, 21, 24, 36, 41, 44) were analysed, values are given as median [95% CI].

**Results:**

FPS improved distinctly after instruction (2.0 [1.48;2.54] vs. 2.83 [2.45;2.95]; (*p* < .001), but not plaque removal (difference PSPI before/after: 0.17 [0.04;0.25] vs. 0.21 [0.13;0.25]; *p* = .112). Plaque removal was not correlated with PSPI.

**Conclusion:**

These findings suggest that even with correct technique, flossing may not substantially reduce plaque levels.

**Clinical relevance:**

The results align with previous studies questioning the efficacy of flossing and highlight the need for further investigation into interdental cleaning approaches.

## Introduction

Interdental hygiene is considered important for maintaining oral health. For removing plaque in the interdental areas many aids are available such as floss, interdental brushes, powered flosser or wood sticks. To date, many studies investigated the efficacy of these aids which may give improvements over toothbrushing alone; however, the certainty of evidence remains low.

Regarding floss, a systematic review [1] indicated a trend towards reduced plaque levels after one month when flossing was added to toothbrushing, but this did not reach significance (standardised mean difference (SMD) −0.42, 95% CI −0.85 to 0.02; seven trials, 542 participants; *p* =.06). Already at the three-month interval the additional effect of flossing decreased markedly (SMD − 0.20, 95% CI −0.36 to −0.04; five trials, 594 participants, *p* =.016), and at six months, flossing was no longer found to have any measurable benefit (SMD − 0.13, 95% CI −0.30 to 0.05; *P* =.53; three trials, 487 participants). If dental flossing can only remove plaque to a limited extent, this also explains the findings of a network meta-analysis that assessed the comparative efficacy of various interdental oral hygiene aids [2] focusing on gingival inflammation. Among 22 trials evaluating 10 different types of devices, ranking probabilities indicated that floss had a near-zero probability of being the best interdental cleaning aid. However, the authors emphasized that the unfavourable results for flossing may be due to its technically demanding applicability and that, when used properly, for example in a professional context (e.g [3])., it could contribute to the prevention of plaque-associated diseases.

Indeed, observational studies show that many subjects have considerable difficulty in using dental floss correctly [4–6]. Frequently, not all interdental spaces are reached by far and floss is only passed over the contact point once and then removed back or pulled through with horizontal movements. Applying floss to the interdental surfaces properly and moving it vertically several times is rarely shown.

Even though little has been published about the best methods and effects of flossing instructions, few studies show that it is possible to improve flossing skills distinctly [4, 6–8]. It therefore seems essential for clinical studies on flossing efficacy to provide participants with detailed instruction and to verify whether the correct use of dental floss has in fact been implemented in the entire dentition. However, whether this was carried out sufficiently well in studies on the effectiveness of dental floss is mostly only reported very poorly.

Thus, the question remains open as to whether the limited effect of dental floss described in the literature is due to inadequate instruction and technical performance or actual uselessness.

The aim of the study was therefore to investigate whether improved flossing skills after instruction are associated with better plaque removal compared to habitual use.

## Participants, materials & methods

The study investigated flossing performance through video observation and monitored plaque levels using an intraoral scanner. It was conducted in accordance with the principles of the Declaration of Helsinki and the principles of Good Clinical Practice. The study was approved by the Ethics Committee of the Justus-Liebig-University Giessen (ref. no. 63/22) prior to commencement. The study was registered in the German clinical trials register (DRKS00034902) and included university students from different faculties, among them dental students from the first semester, who were recruited via notices at central locations and social media.

Participants were eligible for inclusion if they were at least 18 years old, had a sufficiently healthy dentition, provided written informed consent, and were willing to take part in the study. Exclusion criteria were mental and/or physical disability that could limit the subject’s oral hygiene performance, as well as allergies or intolerances to the dental materials used (Mira-2-Ton^®^ solution: sodium benzoate, potassium sorbate, C. 45410, C. 42090; OptraGate lip retractor: styrene-ethylene-butylene-styrene (Ivoclar Vivadent GmbH)). Further exclusion criteria based on anatomical characteristics or factors interfering with standardized plaque assessment and oral hygiene procedures for all teeth: gingivitis with swelling and bleeding (< grade 3 of the Modified Gingival Index [9], tooth mobility > 1 (Grace & Smales Mobility Index [10]), recessions with an extension of more than one third of the root length, cavitated carious lesions, gap dentition, absence of permanent teeth (with the exception of third molars and missing premolars due to orthodontic treatment with gap closure), fillings or crowns involving the proximal surfaces with defects that impede the use of dental floss and represent plaque retention sites, fixed orthodontic appliances.

### Study design

At the first appointment, the inclusion and exclusion criteria were checked, all of the invited subjects were eligible according to in- and exclusion criteria; enrolment was continued until the required sample size was reached. All participants were asked to continue their habitual oral hygiene, but to suspend interdental hygiene measures until the next appointment.

The second appointment (T1) followed after one week. The participants first brushed unsupervised their teeth as usual, using their own toothbrush. In a dental chair, plaque in both jaws was then disclosed with a foam pellet saturated with a plaque revelator (Mira-2-Ton; Hager & Werken, Duisburg, Germany). After the participants had rinsed with tap water, this procedure was repeated in order to achieve the best possible saturation of the plaque with the colouring agent. Then intraoral scans (Carestream CS 3800, Carestream Dental LLC, Atlanta, USA) of both jaws (scan 1) were performed as previously described [11]. The lips and cheeks were held away with a retractor (OptraGate; Ivoclar Vivadent, Schaan, Liechtenstein), and the teeth were kept as dry as possible with an air blower and saliva ejector. The lower jaw was scanned first in order to minimise the revelator being washed out due to saliva accumulation.

The participants then went into another room equipped with a filming device. The video camera (4 K Ultra-HD-Camcorder HC-VX878, Panasonic, Kadoma, Japan) was mounted behind a semi-transparent mirror, which was taped with a transparent colour filter foil (Linaershao, Chengdu, China) to make any red stains on the teeth as invisible as possible. This was to prevent the flossing performance from being affected by the perception of stained plaque. A dispenser with dental floss (Oral-B Essential Floss Mint waxed, Procter & Gamble, Schwalbach am Taunus, Germany) was provided and participants were asked to floss in the way they thought was right and how they usually flossed at home (habitual flossing). Flossing performance was filmed unobserved and without a time limit. The remaining plaque was then recorded with intraoral scans (scan 2) after re-staining as described above.

The participants then watched the instruction video used in a previous study [6]. This video showed in close-up how the floss is correctly guided over the contact point, applied to the mesial and distal tooth surface of the interdental space and moved in vertical direction. In addition, a systematic procedure was demonstrated, starting in the interdental space of the first and second molars in the upper jaw on the right and then successively reaching every subsequent interdental space up to the interdental space of the two molars in the lower jaw on the right. The participants were asked to practice flossing as instructed for one week.

**Fig. 1:**
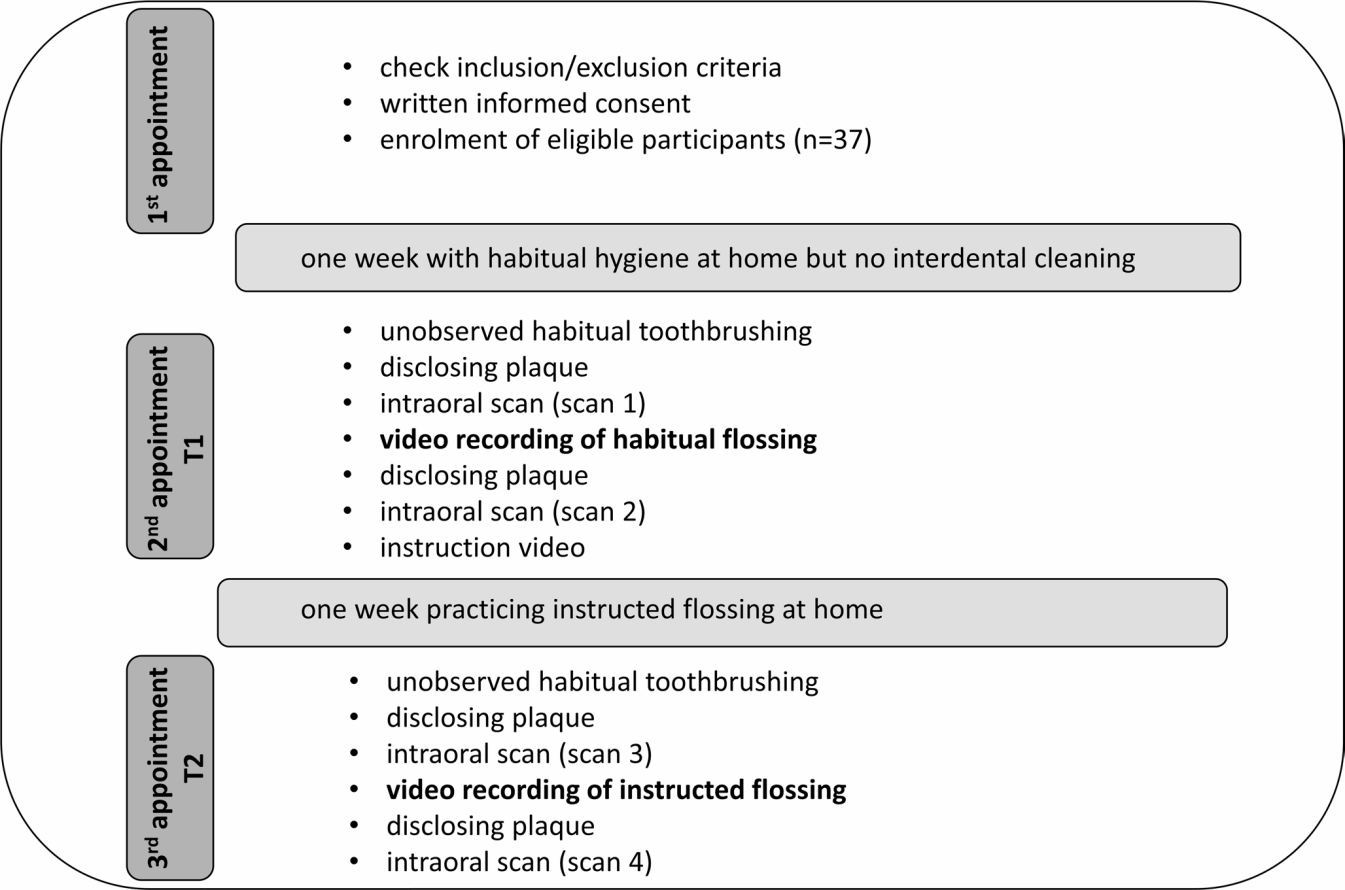
Study flow chart

At the third appointment (T2), the instructed flossing performance were recorded as well as one scan before flossing (scan 3) and one after flossing (scan 4) as described above. Figure 1 shows the study flow.

MK performed the intraoral scans and BB conducted the video analyses. All study procedures were thoroughly trained on before the start of the study, with spot checks carried out throughout.

### Videoanalysis

The videos were analysed after all participants had completed all sessions using special software (Interact version 9.6.1.170, Mangold International, Arnstorf, Germany). All videos were coded exhaustively meaning that every second of the recorded session was annotated for relevant behaviours in slow motion using continuous temporal behavioural sampling. The interdental spaces mesial and distal of the Ramfjord-teeth (16, 21, 24, 36, 41, 44; in total 12 interdental spaces) were analysed.

Criteria of proper flossing performance were taken in part from the Flossing Dexterity Index [7]. These included the number of interdental spaces reached, the movements performed in the interdental spaces, and the adaptation to the mesial and distal sides of the interdental space. An interdental space was defined as reached when the floss passed the contact point, correct movement was coded when vertical movements were performed and correct adaptation when the floss was placed mesially and distally against the tooth surfaces. This was coded dichotomously (yes/no) in each case. A score was calculated from the percentage (decimal numbers) of interdental spaces reached, flossed with vertical movements and with correct adaptation. To describe the overall performance, the individual scores are added together to the flossing performance score (FPS; maximum score 3 = all interdental spaces flossed with correct adaptation and vertical movements). In addition, the duration of flossing was coded and is given in seconds. Duration as well as FPS is given as median [95% CI].

### Plaque assessment

**Fig. 2:**
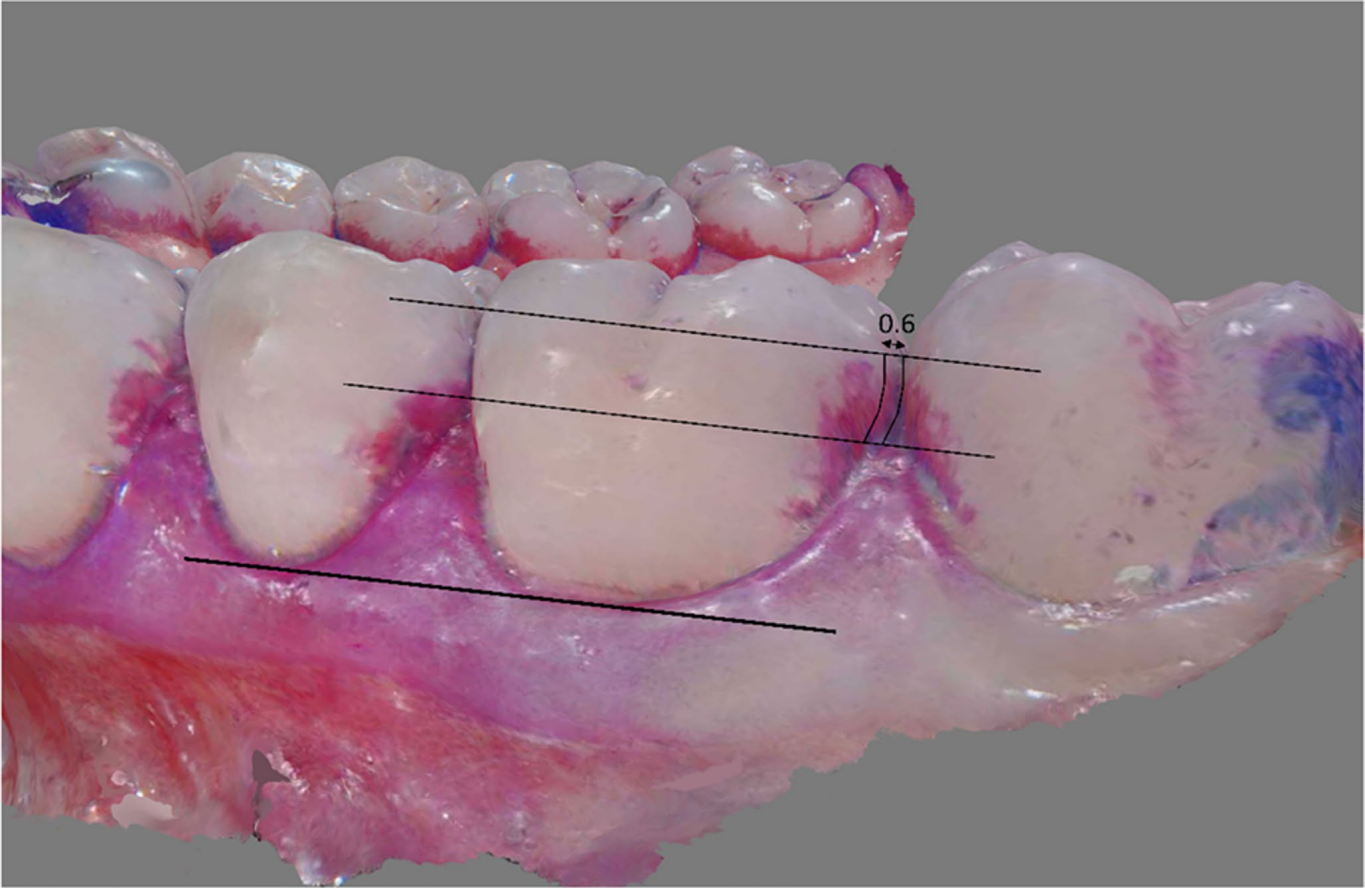
Schematic representation of the constructed examination area using tooth 36 as an example

The scans of the upper and lower jaw of a participant of all four time points were aligned processed using MeshLab software (Version 2022.02) [12]. A region of interest (ROI) was constructed as follows: first, a line was drawn perpendicular to the tooth axis at the level of the gingival margin, which was then shifted parallel at the level of the papilla. The proximal contour of the tooth was then traced up to the contact point and a line parallel towards the centre of the tooth with a dimensionless distance of 0.6 was drawn using the measuring tool (Fig. 2). Since the marking was done on the 3D scan in Meshlab, the anatomical corner points could be accurately assigned at all times. The ROI was transferred identically to all four aligned intraoral scans.

The ROI was then visually assessed with the following scores: 0: no stained plaque visible, 1: < 50% of the ROI covered with stained plaque, 2: ≥ 50% of the ROI covered with stained plaque. Four areas of each Ramfjord tooth (mesial and distal each oral and vestibular) were assessed. A Proximal Surface Plaque Index (PSPI) was calculated as the mean of all scores. Plaque removal was calculated as difference between PSPI before and after flossing, thus, higher values for this difference indicate more plaque removal, i.e., less plaque remaining after flossing.

### Observer agreement

The video evaluation was first trained intensively on videos from a previous study [6] and then the inter-rater agreement was determined on the basis of 10 randomly selected (Research Randomizer 3.0) videos. All kappa values were calculated using the reliability module of INTERACT software, which applies an algorithm for matching codes based on temporal overlap and onset proximity. For codes with duration, events were considered matching if they overlapped by at least 85% and their start times were within a tolerance of 24 frames (0.96 s). For point events (without duration), the overlap criterion was deactivated, and a start tolerance of 2 s was applied. Agreement was defined as the proportion of matching codes within these tolerances relative to the total number of observed codes.

Kappa values for “interdental space reached” were 0.846, for “technique” 0.663 and for “adaptation” 0.486. The intra-rater agreement was determined twice: prior to starting the video analysis and after half of the video analyses had been completed. The videos used for this were also randomly selected. Prior to beginning of the video analysis, the kappa values for “interdental space reached” were 0.881, for “technique” 0.902 and for “adaptation” 0.667. After half of the video analysis, the kappa values were 0.986, 0.863 and 0.708, resp. To determine the repeatability of the assessment of plaque coverage, the four intraoral scans of three randomly selected subjects were analysed a second time. For this purpose, the scans were aligned again and the evaluation areas were reconstructed. The kappa value was calculated with IBM SPSS Statistics version 27 (IBM Deutschland GmbH, Ehningen, Germany) for scoring areas was 0.679.

## Statistics

The sample size calculation was done with G*Power (Version 3.1.9.7) and was based on the improvement in flossing performance that can be achieved through instruction. Data from Radentz et al. [4, 8] and Jung et al. [6] show that instruction can lead to significant improvements compared to baseline values; the proportion of correctly flossed teeth after instruction ranged from approximately 30% [6] to 70% [4, 8] compared to baseline values of between approximately 2% [6, 8] and 12% [4]. For a more conservative estimate, we assumed better flossing performance as the baseline and used the data from [8] after the first and second instruction. With a calculated effect size of 0.48, an alpha of 0.05 and a beta of 0.80, this resulted in a sample size of 37 participants.

Statistics were performed using IBM SPSS. The Kolmogorov-Smirnov test revealed a significant deviation of the Gaussian distribution for most of the data. Therefore, a non-parametric test was used (Wilcoxon-test). Linear regression was used to analyse associations between flossing performance and plaque parameters. Flossing duration as well as FPI and PSPI scores are given as median [95% CI], confidence intervals were obtained by bootstrapping (method of sampling: simple, number of samples: 1000), the level of significance was set at 0.05.

## Results

The group studied consisted of 37 subjects (30 female, 7 male; 23.1 ± 3.2 years), no drop outs occurred.

### Flossing performance

**Fig. 3:**
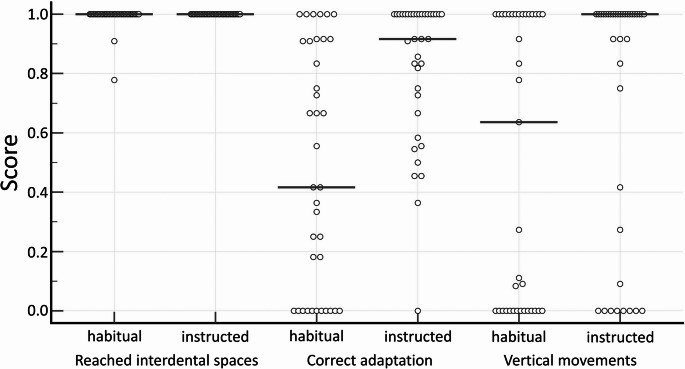
Percentages of correct flossing performance for interdental spaces reached, correct adaptation and correct movements for habitual and instructed flossing by participant. The vertical line indicates the median value. Each circle represents one participant

The FPS for habitual flossing was 2.0 [1.48; 2.54] which increased significantly after instruction (2.83 [2.45; 2.95]; *p* <.001). The score reflects the quality of flossing based on three criteria: percentage of interdental space reached and percentage of interdental spaces with correct floss adaptation and with vertical flossing movements; each rated on a scale from 0 to 1, adding up to a sum sore between 0 (totally imperfect) and 3 (perfect flossing). The distribution of scores for interdental spaces reached, correct adaptation and correct movements for habitual and instructed flossing by participant is given in Fig. 3. Almost all participants had reached all interdental spaces already at T1, but the correct technique was only implemented to a limited extent. For habitual flossing, the score for correct adaptation was 0.42 [0.18; 0.73] and for vertical movements 0.64 [0.0; 1.0], both of which improved significantly after instruction (0.92 [0.83; 1.0], *p* <.001 and 1.00 [0.83; 1.00]; *p* =.012; resp.). However, even after instruction, there were still 8 subjects who did not implement the vertical movement in any interdental space and 5 subjects were only able to present the correct adaptation of the floss in less than half of the interdental spaces.

The time for habitual flossing was 60.3 [48.6; 75.6] s and increased to 89.2 [67.0; 108.7] s after instruction (*p* <.001).

### Plaque assessment

**Fig. 4:**
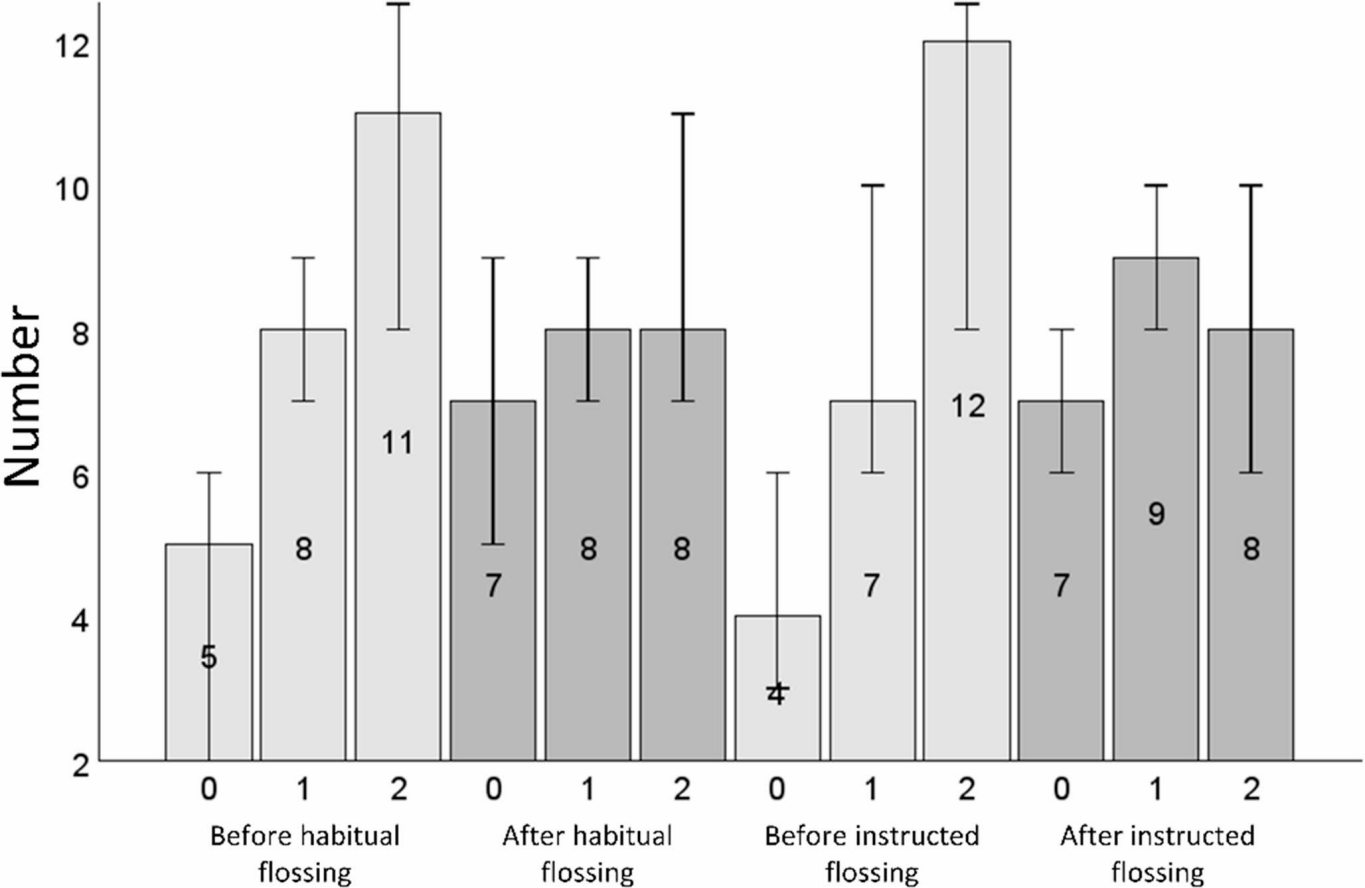
Median [95% CI] number of plaque scores (0: no stained plaque visible, 1: < 50% of the ROI covered with stained plaque, 2: ≥ 50% of the ROI covered with stained plaque) before and after habitual as well as instructed flossing

The PSPI was 1.29 [1.00; 1.42] before habitual and 1.33 [1.13; 1.46] before instructed flossing with no significant difference between them (*p* =.182). Oral sites had higher PSPI values than buccal sites at all time points (all *p* <.01). Overall, the plaque removal (difference PSPI before/after) was 0.17 [0.04; 0.25] for habitual and 0.21 [0.13; 0.25] after instructed use (*p* =.112). There was a very small albeit significant improvement in plaque removal by instruction for the buccal areas (0.08 [0.04; 0.13]) versus 0.17 [0.08; 0.25]; *p* =.002), but not for the oral areas (0.17 [0.08; 0.25] versus 0.17 [0.12; 0.21]; *p* =.332). Values are given in Table 1. Flossing significantly increased the number of plaque-free areas (*p* <.001 for both habitual and instructed flossing) and reduced the number of areas with a score of 2 (*p* <.001 for both habitual and instructed flossing). In contrast, there was no significant change in the number of areas with a score of 1 (*p* =.726 for habitual and *p* =.177 for instructed flossing) (Fig. 4).

**Table 1:** Proximal surface plaque index (PSPI) values (median [95% CI]) before and after flossing overall as well as for vestibular and oral areas, each for habitual and instructed flossing separately. Lower case letters refer to columns, upper case letters to rows. Different letters indicate significant differences

	All areas	Buccal areas	Oral areas
Habitual flossing			
before	1.29 [1.00; 1.42] ^a^	1.25 [0.75; 1.33] ^aA^	1.42 [1.25; 1.58] ^aB^
after	1.04 [0.96; 1.21] ^b^	0.83 [0.67; 1.17] ^bA^	1.17 [1.08; 1.25] ^bB^
Instructed flossing			
before	1.33 [1.13; 1.46] ^a^	1.08 [0.92; 1.33] ^aA^	1.50 [1.25; 1.58] ^aB^
after	1.08 [0.96; 1.13] ^b^	0.92 [0.67; 1.27] ^bA^	1.12 [1.00; 1.33] ^bB^

**Fig. 5:**
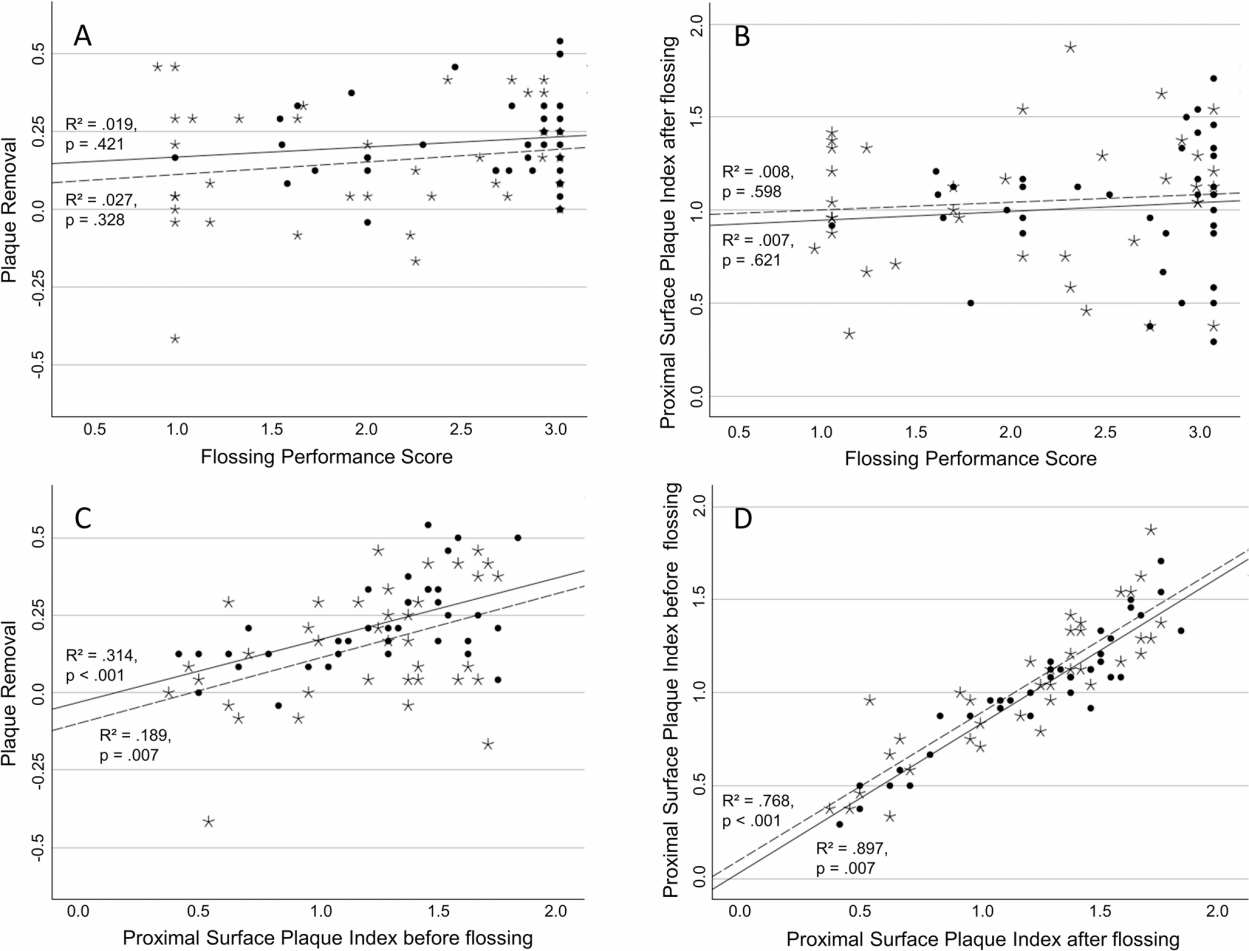
Linear regressions: A: association between flossing performance score and plaque removal (the difference of Proximal Surface Plaque Index before and after flossing); B: association between flossing performance and plaque; C: association between plaque before flossing and plaque removal; D: association between plaque before and after flossing. Asterisks and dashed regression line: habitual flossing; circles and solid regression line: instructed flossing

Flossing performance was not related to plaque scores (difference PSPI before/after) as there was no significant relationship between FPS and PSPI scores after flossing and also no significant relationship between FPS and plaque removal (Fig. 5). Similar was true for the time spent for flossing (habitual flossing: R² = 0.011, *p* =.544; instructed flossing: R² = 0.001, *p* =.825). However, there was a significant relationship between the PSPI score before flossing and plaque removal and a strong relationship between PSPI scores before and after flossing (Fig. 5).

## Discussion

Since it is still unclear whether the limited cleaning effect of the floss is due to insufficient application or to actual uselessness, the present study examined whether, if proper flossing performance is established, effective plaque removal also follows.

To date, there is no clinical method for measuring the effectiveness of interdental cleaning aids within the interdental space, therefore, the area of the tooth surface close to the interdental space is assessed as a surrogate.

Various indices have been used to record plaque in flossing studies [1]; while some index systems appear inappropriate from the outset because they do not consider the approximal area of the teeth as a separate component, for example the Quigley-Hein-Index [13] or its modifications [14], others are more suitable. The Rustogi modified Navy plaque Index [15] for example divides the tooth surface into 9 areas, at least 2 of which can map the plaque coverage in the approximal region. However, it takes a lot of imaginative power to mentally project the complex field division onto a tooth surface and it remains subjective how wide the individual areas are imagined to be and careful training and calibration it required. Furthermore, these areas are usually only assessed dichotomously, i.e. as plaque-free or plaque-containing, which only partially reflects the actual plaque coverage [16]. This means that small changes in plaque accumulation in the relevant areas do not necessarily alter the overall assessment of the area.

In the present study, these methodological problems were avoided by recording plaque on intraoral scans. From the images generated from these scans, a ROI was constructed that was transferred identically after alignment to all scans. Even if the construction was carried out according to specific anatomical structures and defined dimensions, it is still an arbitrary approach. However, own preliminary tests have shown that areas of the width chosen here can be reached if the dental floss is adapted to the proximal surface and pulled slightly around the tooth to perform the vertical movements. Furthermore, these areas were not only assessed dichotomously but in three degrees, which allows a more differentiated assessment of the cleaning result [16]. Images from intraoral scans had also been used in another study that investigated the cleaning efficacy of interdental brushes [17] which were analysed using a planimetric method comparing areas of plaque-covered sites before and after the use of interdental brushes. However, it is not specified how the relative proportions were calculated and how reliable the method was.

The flossing performance was recorded with video observations and assessed based on the flossing dexterity index [7] as in our previous studies [5, 6]. The relevant parameters were whether the interdental space was reached at all and whether the floss was moved with vertical movements on both interdental tooth surfaces. The correct performance was communicated with an instructional video, which we had already used in a previous study and had achieved the intended instruction effect [6]. As our intention was to achieve a substantial instruction effect, a group of university students were included, who were expected to have good comprehension and motor skills.

Even though the included group could have been assumed to have good oral hygiene, many areas still exhibited the highest plaque score even after toothbrushing. However, it was also observed that some interdental spaces were already plaque-free at T1, suggesting that effective cleaning of these areas is in principle achievable by toothbrushing alone. With habitual flossing, almost all participants were able to reach all interdental spaces, which indicates a certain familiarity with the use of dental floss. Perhaps those who were already experienced at flossing in their daily lives were also more likely to have registered for the study. However, only about half were able to present the proper technique. Thus, the participants in the present study performed better than in earlier study [5] in terms of interdental spaces reached, but not in terms of flossing technique. After habitual flossing, the amount of plaque was reduced in the order of about 13%, with an improvement only in the buccal, but not the oral surfaces; areas with substantial plaque coverage (50% of the area) were reduced from 46 to 33%, but only 29% were plaque-free.

Instruction can significantly improve flossing performance distinctly [4, 6, 8], which was also the case in the present study. After instruction, the flossing performance score increased significantly as did the time the participants spent flossing. Contrary to our assumptions, however, this improvement in flossing performance did not lead to a relevant improvement in the cleaning effect. Plaque scores were reduced by 16%, which is only 3% more than after habitual flossing. Further, there was no relationship between flossing performance and plaque amounts, and there was a remarkable range of plaque scores even in subjects who achieved the highest flossing performance score. Instead, a considerable relationship between plaque sores before and after flossing persisted. This is all the more unexpected given that the participants not only demonstrated notable flossing skills, but also spent a lot of time (around 7.5 s per interdental space).

The present study supports findings from an early observational study investigating different types of flossing devices in schoolchildren [7]. Mechanical plaque removal through flossing contributed little to plaque reduction in young children and there was also no correlation between flossing dexterity and plaque removal suggesting that even when flossing skills improve, plaque reduction does not necessarily follow.

Thus, the present results appear to refute the initial assumption that the lack of effectiveness of floss may be due to inadequate application rather than actual uselessness.

However, the presence of plaque residues on the floss after use suggests that some degree of plaque removal does occur during flossing. This may be successful if the plaque masses are thick and have a rather low internal adhesion. Thinner biofilms, on the other hand, could be much more difficult to remove. Structured biofilms represent a microbial community embedded in an extracellular matrix [18] and can exhibit considerable adhesion strength to the surfaces they cover. For example, on titanium surfaces adhesion strength values of *Strep. mutans* biofilms of around 300 MPa were measured [19] and it is interesting that the presence of sugar modulates the adhesion strength considerably [20]. It is also the question of how disrupted biofilm can be removed from the tooth surface. Some of the biofilm mass might stick to the floss, but other parts might simply be pushed back and forth and then remain on the tooth surface. This could also explain why in a few cases we found more stained plaque after flossing than before.

Nonetheless, there may be benefits to flossing that are not necessarily related to plaque removal. In this context, it is interesting to note that interdental brushes are probably more effective at removing plaque than floss, but the effect on gingivitis is about the same for both devices [1, 21]. This suggests that the mechanical disintegration of the biofilm may have an impact independent of the cleaning effect. In a twin study [22] it was shown that flossing significantly reduced the abundance of periodontal pathogens and cariogenic bacteria compared to the non-flossing group. This, however, was not confirmed in a more recent study showing that antimicrobial mouth rinses significantly reduced microbial diversity, species richness, and bacterial abundances but not brushing alone or brushing with flossing [23]. It is, however, important to recognize that plaque-associated conditions such as dental caries and periodontal disease are not only determined by the quantity of plaque present, but are also significantly influenced by a complex interplay of behavioural factors and the host’s immune response [24–27]. In any case, many questions remain unanswered after the present study and this could be a starting point for further studies investigating the mechanical effects of dental floss on biofilms in more detail.

The present study certainly has limitations, especially because it was a short-term observation with only one instruction. Although the participants showed a good flossing performance overall, the effects might have been clearer if the instructions had been more frequent and the practice times longer. Future studies could investigate whether alternative flossing techniques or devices lead to improved outcomes. Furthermore, the results cannot simply be transferred to other groups of people, for example, groups of people of a similar age but from other socio-economic contexts and parameters related to gingival health were not obtained. Ultimately, as in all other studies, conclusions about plaque levels within the interdental space can only be drawn indirectly.

Nevertheless, the results point to the need for further research beyond traditional flossing techniques, for example whether more effective movements are conceivable or whether the force used to move the dental floss along the tooth surfaces plays a role. A method for quantifying plaque within the interdental space would probably provide new insights in the context of interdental hygiene.

## Conclusion

We can conclude that even a high level of flossing performance does not contribute to effective plaque reduction in the area of the tooth surfaces close to the interdental space.

## Data Availability

Data is provided within the manuscript.
